# Prognostic factors for squamous cervical carcinoma identified by competing-risks analysis: A study based on the SEER database

**DOI:** 10.1097/MD.0000000000030901

**Published:** 2022-09-30

**Authors:** Chengfeng Hu, Junyan Cao, Li Zeng, Yao Luo, Hongyuan Fan

**Affiliations:** a Department of Obstetrics and Gynecology, The Second Affiliated Hospital of Guizhou University of Traditional Chinese Medicine, Guiyang, China; b Guizhou University of Traditional Chinese Medicine, Guiyang, China.

**Keywords:** cause-specific, cervical cancer, competing-risks model, prognostic factors, sub-distribution

## Abstract

Cervical cancer has a high incidence of malignant tumors and a high mortality rate, with squamous cervical carcinoma (SCC) accounting for 80% of cases. A competing-risks model is recommended as being more feasible for evaluating the prognosis and guiding clinical practice in the future compared to Cox regression. Data originating from the Surveillance, epidemiology, and end results (SEER) database during 2004 to 2013 were analyzed. Univariate analysis with the cumulative incidence function was performed to assess the potential risk of each covariate. Significant covariates (*P *< .05) were extracted for inclusion in a Cox regression analysis and a competing-risks model that included a cause-specific (CS) hazard function model and a sub-distribution (SD) hazard function model. A total of 5591 SCC patients met the inclusion criteria. The three methods (Cox regression analysis, CS analysis, and SD analysis) showed that age, metastasis, American Joint Committee on Cancer stage, surgery, chemotherapy, radiation sequence with surgery, lymph node dissection, tumor size, and tumor grade were prognostic factors affecting survival in patients with SCC. In contrast, race and radiation status were prognostic factors affecting survival in the Cox regression and CS analysis, but the results were different in the SD analysis. Being separated, divorced, or widowed was an independent prognostic factor in the Cox regression analysis, but the results were different in the CS and SD analyses. A competing-risks model was used as a new statistical method to more accurately identify prognostic factors than conventional Cox regression analysis leading to bias in the results. This study found that the SD model may be better suited to estimate the clinical prognosis of a patient, and that the results of an SD model analysis were close to those of a CS analysis.

## 1. Introduction

Cervical cancer is cancer with a high incidence of malignant tumors in the female reproductive system, ranking second only to breast cancer, and it is the fourth leading cause of female malignancy, thereby representing a major threat to the health and life of women. It is reported that the average number of new cases annually is about 500,000 with about 250,000 deaths, and the number of new cases in China each year is more than 10,000 corresponding to about 25% of the global reported new cases of cervical cancer.^[1,2]^ In 1992, the WHO announced that high-risk human papillomavirus (HPV) infection was the primary factor contributing to cervical cancer. In 1995 the International Cancer Society also proposed that HPV infection, especially long-term persistent infection of high-risk HPV, was the main cause of cervical intraepithelial neoplasia and the further development into cervical cancer. A meta-analysis showed that high-grade squamous intraepithelial lesions infected with HPV 16, 18, and 45 are more likely to develop into squamous cervical carcinoma (SCC) than that infected with other sub-types of HPV.^[3]^

The use of a bivalent vaccine against HPV has greatly reduced the incidence of cervical cancer, but this remains the most commonly diagnosed type of cancer at present in both developed and developing countries.^[4]^ A European randomized controlled trial indicated that the application of HPV-based cervical screening could reduce the number of women, who develop invasive cancers by 60% to 70%,^[5]^ and so it should be promoted among young women starting from the age of 30 years followed by screening every 5 years.

While the cause of cervical cancer is clear, screening and prevention apply to cervical cancer in the nonoccurrence stage. However, for patients who already have cervical cancer, there are many factors interfering with its progression that influence the cancer mortality rate. Some of the factors that have been analyzed using conventional Cox regression include basic patient characteristics (e.g., age, stage, race, and marital status), tumor characteristics (e.g., tumor stage, tumor size, invasion, metastasis, and lymph node [LN] status), and treatments (e.g., surgery, chemotherapy, radiation, and lymph node dissection [LND]).^[6–13]^ Overall, tumor characteristics and treatments are closely related to the prognosis of the disease. However, the applied treatment is largely determined by the nature of the tumor. Surgery and radiation therapy are major treatments for cervical cancer, with chemotherapy being an adjunctive systemic therapy. Surgery and/or radiation are often applied to patients at cervical cancer stages IA2–IIA, with radical hysterectomy combined with regional lymphadenectomy being a conventional treatment for those people, and ovarian conservation of hysterectomy having positive effects in decreasing the all-cause mortality. However, for locally advanced cervical cancer, concurrent chemoradiotherapy has been a better choice. These conclusions were basically obtained from Cox regression analyses, and there have been some deviations. The present study found that a competing-risks model has its own advantages in analyzing the prognosis.

In medical practice, a longitudinal analysis does not always identify only events of interest to researchers, but also some outcomes that are not of interest. There is a competing relationship between events of interested and uninterested outcomes. For such data with competing risks, previous approaches have involved defining competing events as censored data as well. If competing risks are ignored, the traditional univariate analysis method of Kaplan–Meier marginal regression will overestimate the cumulative mortality, and the traditional Cox multivariate regression analysis may provide only poor estimates of the hazard ratio (HR). The statistics of the competing-risks model could not be analyzed in previous versions of SAS software, but now the R software provides an additional program package (called “cm prsk”) that is widely used in the sub-distribution hazard function competing-risks model. Version 9.4 of SAS uses the SD model, also called the cumulative incidence function (CIF) regression model or Fine-Gray model, in conjunction with the cause-specific (CS) hazard function model to better assess the prognosis of a disease in a competing-risks model.^[14]^ A CS model may be better suited to addressing etiological questions, whereas an SD model might be better suited to estimate the clinical prognosis of a patient.^[15]^ Some studies have indicated that using SD and CS models simultaneously is generally the most rigorous scientific approach to analyzing competing-risks data.^[16]^

In this study, the primary endpoint of concern was death due to squamous cervical carcinoma (DCC), and a competing event was death due to other causes (DOC). SAS statistical software (version 9.4) was used to assess the survival of patients with SCC with the aim of identifying more accurate and reliable risk factors for DCC, in order to guide clinical practice.

## 2. Materials and methods

### 2.1. Data source

The analyzed data originated from the Surveillance, epidemiology, and end results (SEER) database that was been established by the National Cancer Institute. This database is supported by the Surveillance Research Program in the National Cancer Institute Division of Cancer Control and Population Sciences, and includes information on cancer incidence, treatment, and survival for approximately 30% of the US population. The SEER database contains data on cancer cases from various locations and sources throughout the US from 1973, which are comparable with the general population characteristics of the US. SEER data can be used to address multiple topics such as examining the stage at diagnosis according to race/ethnicity, determining trends and incidence rates for various cancer sites over time, and calculating survival according to the stage at diagnosis, age at diagnosis, and tumor grade or size. We utilized the Incidence-SEER 18 Regs Research Data + Hurricane Katrina Impacted Louisiana Cases, Nov 2017 Sub (1973–2015 varying) database. SEER research data are publicly available, and all patient information is de-identified, which meant that institutional review board approval was not required.

### 2.2. Patient selection

**Figure 1.**
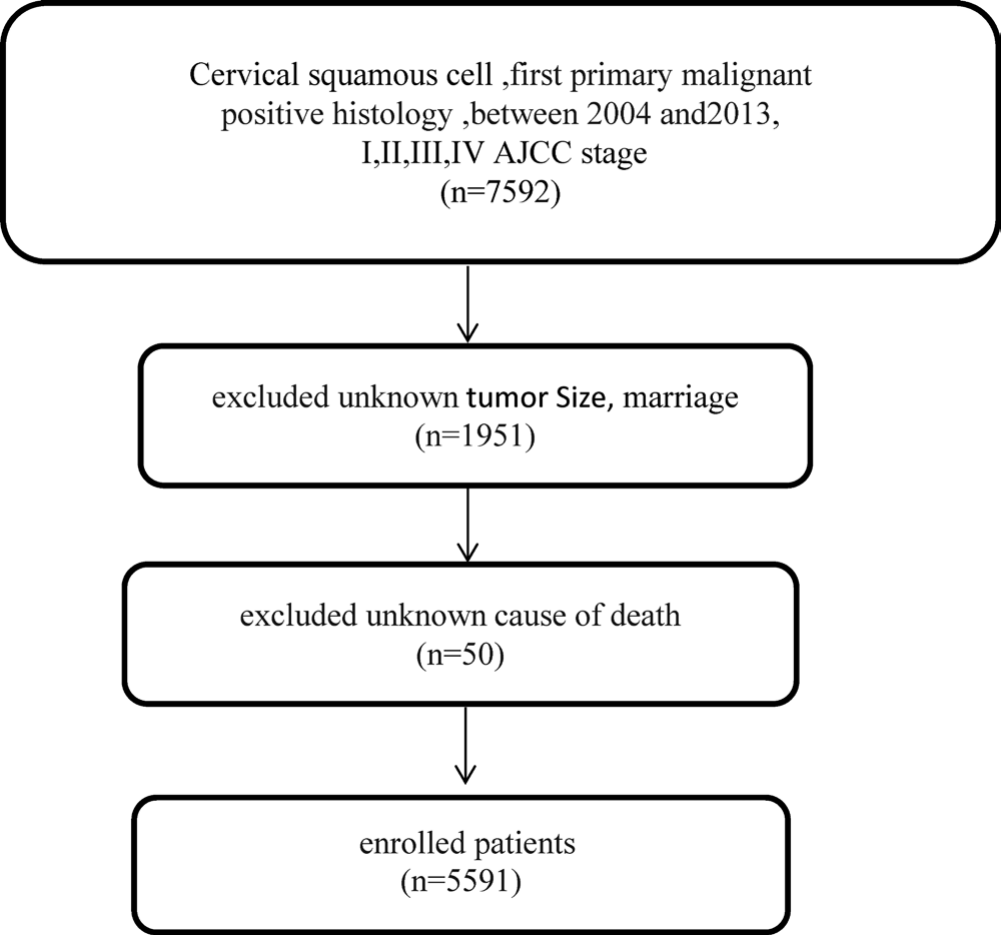
Diagram for data abstraction and exclusion.

We identified 5591 patients diagnosed with SCC between 2004 and 2013. The endpoint of 2013 was selected to ensure that the follow-up was adequate in all of the included women. The following demographic and clinical variables were extracted: age, race, marital status, LN metastasis stage (according to the third edition of the American joint committee on cancer [AJCC]), positive lymph node, metastasis, surgery, chemotherapy, radiation, radiation sequence with surgery (RSS), LND, tumor size, and tumor grade. In the analysis we regrouped the AJCC stages into Ia, Ib, IIa, IIb, III, and IV, and categorized grades I, II, III, and IV as well-differentiated, moderately differentiated, poorly differentiated, and undifferentiated, respectively. The inclusion criteria were as follows: diagnosed between 2004 and 2013; pathological sites including the endocervix, exocervix, overlapping lesion of cervix uteri, and cervix uteri; positive histology with squamous cell carcinoma; presence of a single primary tumor; being the first malignant primary tumor. The exclusion criteria were as follows: autopsy or death certificate; positive histology with adenocarcinoma, gland scale cancer, endometrial carcinoma, and adenoid basal cell carcinoma; unknown tumor grade; unknown marital status; unknown AJCC stages; unknown cause of death; or unknown tumor size. The flow chart of the study is shown in Figure 1.

### 2.3. Clinicopathological factors

We extracted the following 14 factors from the SEER database: age, race, marital status, AJCC stage, LN metastasis, surgery, chemotherapy, radiation, RSS, LND, tumor size, and tumor grade. Age was a continuous variable, while the other variables were categorical variables. Race was divided into three types: white, black, and other (American Indian/AK Native, Asian/Pacific Islander). Marital status was divided into three types: married, single, and other (separated, divorced, or widowed). LN metastasis was classified into two types: yes and no. Chemotherapy, radiation, and LND are classified into two types: yes and no/unknown. The types of surgery were yes, no, and unknown. Tumor size was classified into ≤40 mm, 40 mm to 100 mm, and ≥100 mm. RSS included radiation prior to surgery (RPS), intraoperative radiation, radiation after surgery (RAS), and no/unknown. The follow-up results were divided into three conditions: alive, DCC, and DOC.

### 2.4. Statistical analysis

All of the statistical analyses were performed using SAS statistical software (version 9.4, SAS Institute). A univariate analysis was performed with the CIF to assess the potential prognostic contribution of each covariate, and obtain the value from Gray’s test and the cumulative incidence rate at each time point. Significant covariates (*P *< .05) were extracted for inclusion in multivariate Cox regression, CS, and SD analyses; an SD analysis is also called Fine-Gray competing-risks regression. Multivariate Cox regression analysis was performed to identify covariates associated with an increased all-cause mortality. SCC-specific mortality was assessed CS and Fine-Gray competing-risks regression.^[14]^ *P *< .05 was considered statistically significant.

## 3. Results

### 3.1. Patient characteristics

A total of 5591 patients with SCC met the inclusion criteria (Table 1). At the last follow-up, 2671 patients were still alive: there were 1578 DCCs and 1342 DOCs. The median age was 48.0 years old (range = 19–98 yr old) for all patients and 50.0 years (range = 21–98 yr old) for DCCs; the corresponding median follow-up times were 47.0 months (range = 0–43 mo) and 16.0 months (range = 0–137 mo), respectively. The median age of all patients was similar to that of DCC patients, whereas the median follow-up time was shorter for DCC patients than for all patients. Overall, the incidence rates were highest among patients who were white, with no metastasis, with negative regional LNs, who underwent surgery, who received chemotherapy, who received radiotherapy, had a no/unknown RSS status, had no LND, and were married, at 73.63%, 91%, 71.74%, 54.03%, 57.99%, 68.56%, 70.41%, 58.95%, and 43.01%, respectively.

**Table 1:** Characteristics and demographics of patients with squamous cervical carcinoma.

Prognostic factors	Classification	All (%)	Death due to cervical cancer
n		5591	1578
Age		Mean 49.56; median 48.0; range 19-98	Mean 51.96; median 50.0; range 21-98
ST		Mean 54.84; median 47.0;	Mean 21.97; median 16.0;
Race			
	White	4117 (73.63)	1115 (70.66)
	Black	905 (16.19)	310 (19.64)
Metastasis	Other Yes No	569 (10.18) 503 (9.00) 5088 (91.00)	153 (9.70) 388 (24.59) 1190 (75.41)
LN			
	Yes	1580 (28.26)	716 (45.37)
	No	4011 (71.74)	862 (54.63)
AJCC stage			
	Ia	585 (10.46)	17 (1.08)
	Ib	1757 (31.43)	211 (13.37)
	IIa	267 (4.78)	78 (4.94)
	IIb	755 (13.50)	203 (12.86)
	III	1611 (28.81)	613 (38.85)
	IV	616 (11.02)	456 (28.90)
Surgery			
	Yes	3021 (54.03)	449 (28.45)
	No	2539 (45.41)	1114 (70.60)
	Unknown	31 (0.56)	15(0.95)
Chemotherapy			
	Yes	3242 (57.99)	1163 (73.70)
	No/Unknown	2349 (42.01)	415 (26.30)
Radiation			
	Yes	3833 (68.56)	1333 (84.47)
	No/Unknown	1758 (31.44)	245 (15.53)
Rss			
	No/Unknown	3937 (70.41)	1160 (73.51)
	RPS	126 (2.25)	45 (2.85)
	IR	2 (0.04)	1 (0.06)
	RAS	1496 (26.76)	358 (22.69)
	RBAS	30 (0.54)	14 (0.89)
LND			
	Yes	2295 (41.05)	354 (22.43)
	No/Unknown	3296 (58.95)	1224 (77.57)
Marriage			
	Yes	2410 (43.10)	533 (33.78)
	None	1708 (30.55)	546 (34.60)
	Other	1473 (26.35)	499 (31.62)
Size			
	≤4 cm	2836 (50.72)	412 (26.11)
	4–10 cm	2569 (45.95)	1047 (66.35)
	≥10 cm	186 (3.33)	119 (7.54)
Grade			
	Grade I	371 (6.64)	46 (2.92)
	Grade II	2536 (45.36)	639 (40.49)
	Grade III	2604 (46.57)	873 (55.32)
	Grade IV	80 (1.43)	20 (1.27)

AJCC = American joint committee on cancer stage, Grade I = well differentiated, Grade II = moderately differentiated, Grade III = poorly differentiated, Grade IV = undifferentiated, IR = Intraoperative radiation, LND = lymph node dissection, RAS = radiation after surgery, RBAS = Radiation before and after surgery, RPS = radiation prior to surgery, RSS = radiation sequence with surgery, ST = survival time.

### 3.2. Univariate analysis

**Figure 2.**
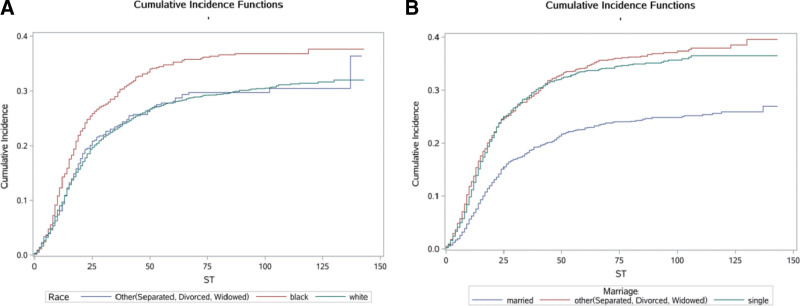
Cumulative risk curves of cause-specific mortalities for demographic characteristics. (A) Race. (B) Marriage.

**Figure 3.**
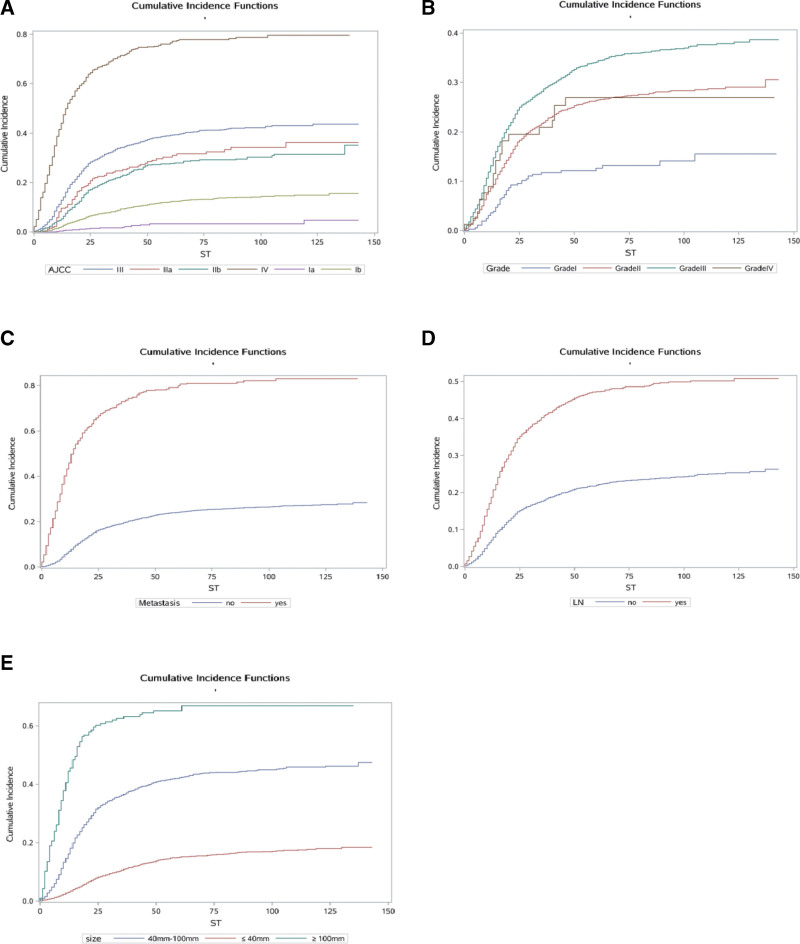
Cumulative risk curves of cause-specific mortalities for tumor characteristics. (A) FIGO stage. (B) Grade. (C) Metastasis (D) Lymph nodes. (E) Tumor size.

**Figure 4.**
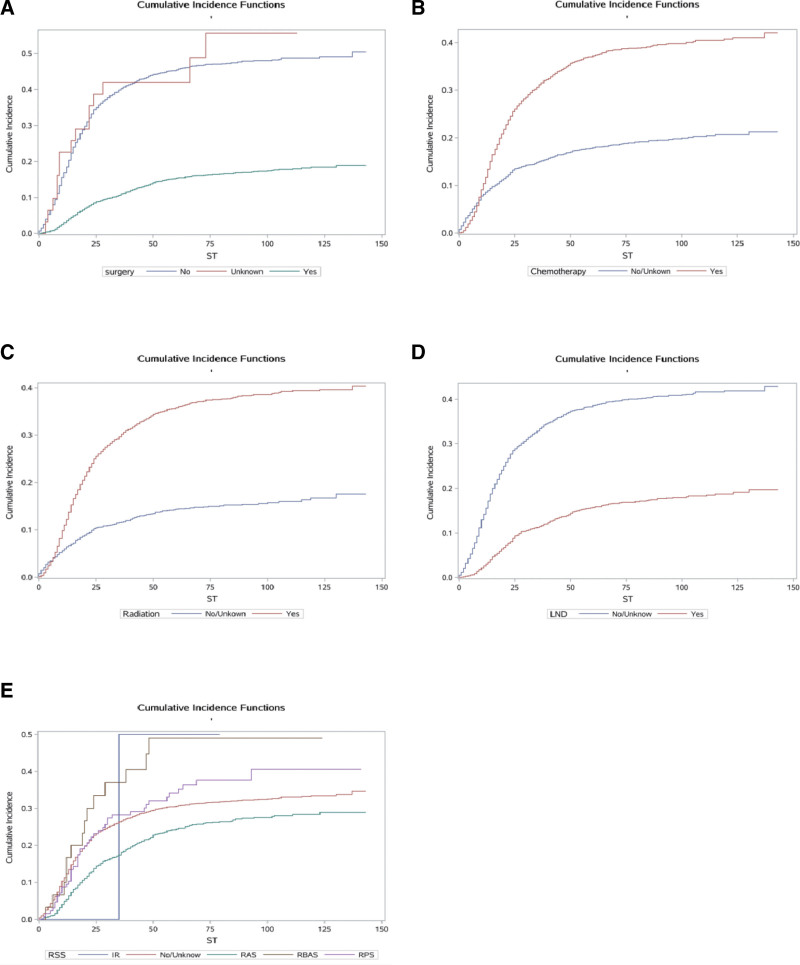
Cumulative risk curves of cause-specific mortalities for treatment. (A) Surgery. (B) Chemotherapy. (C) Radiation. (D) Lymph node dissection. (E) Radiation sequence with surgery.

We calculated the crude CIFs for all prognostic factors. Table 2 lists the significantly influenced (*P *< .05) CIFs for cause-specific mortality according to Gray’s test. The results showed that age, race, marital status, AJCC stage, LN metastasis, surgery, chemotherapy, radiation, RSS, LND, tumor size, and tumor grade were statistically significant in the crude CIF for cancer mortality, and so all of these factors were extracted for inclusion in the multivariate analysis. The cumulative incidence curves are plotted in Figure 2–4 (including demographic characteristics, tumor characteristics and treatment). Meanwhile, the cumulative incidence rates at 1, 3, and 5 years were calculated, and are presented in Table 2.

**Table 2:** Univariate analysis of prognostic factors in patients with cervical squamous cell carcinoma.

Prognostic factors	Classification	Gray’s test	*P* value	12-mo CIF	36-mo 58-mo CIF CIF
Age		231.712	<.0001		
Race		16.2374	.0003		
	White			0.09709	0.23314 0.27901
	Black			0.14304	0.29755 0.35266
	Other			0.09358	0.23680 0.28104
Metastasis		969.150	<.0001		
	Yes			0.45899	0.73330 0.79912
LN	No Yes No	344.592	<.0001	0.06905 0.18705 0.07136	0.19591 0.24129 0.40775 0.47265 0.17924 0.21978
AJCC stage		1432.70	<.0001		
	Ia			0.00442	0.01853 0.03323
	Ib			0.02048	0.08431 0.12152
	IIa			0.09634	0.24725 0.30953
	IIb III IV			0.05277 0.12774 0.44035	0.21874 0.27745 0.33478 0.39147 0.70193 0.76582
Surgery		636.482	<.0001		
	Yes No			0.03523 0.18457	0.10931 0.15352 0.40192 0.45404
	Unknown			0.25806	- 0.48790
Chemotherapy		206.820	<.0001		
	Yes No/Unknown			0.11709 0.08616	0.31090 0.37150 0.15032 0.17940
Radiation		242.643	<.0001		
	Yes			0.12228	0.30161 0.35799
RSS LND Marriage Size Grade	No/Unknown No/Unknown RPS IR RAS RBAS Yes No/Unknown Yes None Other ≤4 cm 4–10 cm ≥10 cm Grade I Grade II Grade III	32.6047 346.393 85.8788 676.798 86.9910	<.0001 <.0001 <.0001 <.0001 <.0001	0.06408 0.12309 0.09524 - 0.05486 0.16667 0.02992 0.15578 0.06865 0.12559 0.13752 0.03016 0.16166 0.44454 0.03591 0.08672 0.13150	0.111620 0.14362 0.26592 0.30607 0.28705 0.03536 - - 0.18103 0.24400 0.40500 - 0.11449 0.15680 0.33408 0.38547 0.18624 0.22864 0.29001 0.33609 0.28547 0.34233 0.10909 0.15025 0.36595 0.42227 0.63140 0.66820 0.11743 0.12643 0.21937 0.26387 0.28686 0.34100
	Grade IV			0.10250	0.20904-

AJCC = American joint committee on cancer stage, CIF = cumulative incidence function, Grade I = well differentiated, Grade II = moderately differentiated, Grade III = poorly differentiated, Grade IV = undifferentiated, IR = Intraoperative radiation, LN = lymph node, LND = lymph node dissection, RAS = radiation after surgery, RBAS = radiation before and after surgery, RPS = radiation prior to surgery, RSS = radiation sequence with surgery, ST = survival time.

### 3.3. Multivariate analysis

The significant prognostic factors extracted in the univariate analysis were included in the multivariate Cox regression, CS, and SD analyses (Table 3). There were some differences in the results for these three methods, with each prognostic factor showing differences in their stratification across the methods. The multivariate Cox regression analysis indicated that age (*P *< .001, HR = 1.018), race (*P *= .0019, HR = 0.832), metastasis (*P *< .001, HR = 2.998), stage Ib (*P *< .001, HR = 3.147), stage IIa (*P *< .001, HR = 6.222), stage IIb (*P *< .001, HR = 6.066), stage III (*P *< .001, HR = 9.208), stage IV (*P* < .001, HR = 27.09), surgery (*P *< .001, HR = 0.379), chemotherapy (*P *< .001, HR = 0.606), radiation (*P* = .005, HR = 0.760), RPS (*P* < .001, HR = 2.411), RAS (*P* < .001, HR = 1.748), radiation before and after surgery (RBAS) (*P* < .001, HR = 3.072), LND (*P* < .001, HR = 0.507), being married (*P* < .001, HR = 0.788), other marital status (*P* < .001, HR = 1.258), size = 4–10 cm (*P* < .001, HR = 1.639), size ≥10 cm (*P* < .001, HR = 2.619), grade II (*P* = .0054, HR = 1.424), and grade III (*P* < .001, HR = 1.742) were independent prognostic factors affecting survival.

**Table 3:** Multivariate analysis of prognostic factors in patients with cervical squamous cell carcinoma.

	Cox regression analysis		SD model analysis		CS model analysis	
Prognostic factors<?Char=Text?>	*P* value<?Char=Decimal?>	HR (95%CI)<?Char=Mixed?>	*P* value<?Char=Decimal?>	HR (95%CI)<?Char=Mixed?>	*P* value<?Char=Decimal?>	HR (95%CI)<?Char=Mixed?>
**Age**	<.001	1.018 (1.015–1.022)	.0014	1.006 (1.002–1.010)	<.001	1.009 (1.005–1.012)
**Race**	-	-	-	-	-	-
White	.0019	0.832 (.0740–.0934)	.727	0.881 (0.767–1.012)	.0299	0.866 (0.761–0.986)
Black	ref	ref	ref	ref	ref	ref
**Metastasis**						
Yes	<.001	2.998 (2.608–3.447)	<.001	3.212 (2.721–3.790)	<.001	3.283 (2.835–3.801)
No	ref	ref	ref	ref	ref	ref
**AJCC stage**						
Ia (ref)	ref	ref	ref	ref	ref	ref
Ib	<.001	3.147 (2.200–4.501)	<.001	4.088 (2.500–6.682)	<.001	4.100 (2.501–6.722)
IIa	<.001	6.222 (4.178–9.266)	<.001	10.99 (6.521–18.52)	<.001	10.995 (6.499–18.600)
IIb	<.001	6.066 (4.220–8.719)	<.001	9.665 (5.902–15.83)	<.001	9.766 (5.948–16.036)
III	<.001	9.208 (6.493–13.058)	<.001	15.51 (9.598–25.05)	<.001	15.945 (9.843–25.829)
IV	<.001	27.09 (19.01–38.617)	<.001	49.14 (30.28–79.76)	<.001	52.531 (32.319–85.384)
**Surgery**						
Yes	<.001	0.379 (0.320–0.449)	<.001	0.406(0.331–0.498)	<.001	0.378(0.314–0.455)
No(ref)	ref	ref	ref	ref	ref	ref
**Chemotherapy**						
Yes	<.001	0.606 (0.532–0.689)	<.001	0.693 (0.587–0.819)	<.001	0.639 (0.553–0.738)
No/Unknown	ref	ref	ref	ref	ref	ref
**Radiation**						
Yes	.005	0.760 (0.628–0.920)	.323	0.766 (0.600–0.978)	.0058	0.745 (0.604–0.918)
No/Unknown	ref	ref	ref	ref	ref	ref
**RSS**						
No/Unknown	ref	ref	ref	ref	ref	ref
RPS	<.001	2.411 (1.776–3.275)	<.001	2.295 (1.580–3.313)	<.001	2.335 (1.666–3.272)
RAS	<.001	1.748 (1.459–2.094)	<.001	1.778 (1.430–2.211)	<.001	1.827 (1.499–2.225)
RBAS	<.001	3.072 (1.769–5.337)	<.001	3.559 (1.989–6.368)	<.001	3.638 (2.082–6.357)
**LND**						
Yes	<.001	0.507 (0.439–0.586)	<.001	0.518 (0.440–0.611)	<.001	0.497 (0.423–0.585)
No/Unknown	ref	ref	ref	ref	ref	ref
**Marriage**						
Yes	<.001	0.788 (0.704–0.884)	<.001	0.726 (0.639–0.826)	<.001	0.725 (0.641–0.820)
No (ref)	ref	ref	ref	ref	ref	ref
Other	<.001	1.258 (1.124–1.408)	.5268	1.044 (0.914–1.191)	.2540	1.075 (0.950–1.271)
**Size**						
≤4 cm (ref)	ref	ref	ref	ref	ref	ref
4–10 cm	<.001	1.639 (1.459–1.842)	<.001	1.936 (1.682–2.228)	<.001	1.950 (1.706–2.227)
≥10 cm	<.001	2.619 (2.129–3.221)	<.001	3.050 (2.338–3.979)	<.001	3.303 (2.645–4.124)
**Grade**						
Grade I (ref)	ref	ref	ref	ref	ref	ref
Grade II	.0054	1.424 (1.110–1.826)	.0005	1.658 (1.247–2.205)	.0011	1.653 (1.233–2.235)
Grade III	<.001	1.742 (1.360–2.232)	<.001	1.979 (1.490–2.629)	<.001	2.007 (1.487–2.709)
Grade IV	.2219	1.344 (0.836–2.160)	.836	1.633 (0.937–2.845)	.0417	1.730 (1.021–2.931)

AJCC = American joint committee on cancer stage, CS = cause-specific, Grade I = well differentiated, Grade II = moderately differentiated, Grade III = poorly differentiated, Grade IV = undifferentiated, HR = hazard ratio, IR = Intraoperative radiation, LND = lymph node dissection, RAS = radiation after surgery, RBAS = Radiation before and after surgery, RPS = radiation prior to surgery, RSS = radiation sequence with surgery, SD = sub-distribution, ST = survival time.

The SD model showed that age (*P* = .0014, HR = 1.006), metastasis (*P* < .001, HR = 3.212), stage Ib (*P* < .001, HR = 4.008), stage IIa (*P* < .001, HR = 10.99), stage IIb (*P* < .001, HR = 9.665), stage III (*P* < .001, HR = 15.51), stage IV (*P* < .001, HR = 49.14), surgery (*P* < .001, HR = 0.46), chemotherapy (*P* < .001, HR = 0.693), RPS (*P* < .001, HR = 2.295), RAS (*P* < .001, HR = 1.778), RBAS (*P* < .001, HR = 3.559), LND (*P* < .001, HR = 0.518), being married (*P* < .001, HR = 0.0726), size = 4–10 cm (*P* < .001, HR = 1.936), size ≥10 cm (*P* < .001, HR = 3.050), grade II (*P* = .0005, HR = 1.658), and grade III (*P* < .001, HR = 1.979) were statistically significant for the prognosis of SCC.

The CS model indicated that age (*P* < .001, HR = 1.009), race (*P* = .0299, HR = 0.866), metastasis (*P* < .001, HR = 3.282), stage Ib (*P *< .001, HR = 4.100), stage IIa (*P* < .001, HR = 10.955), stage IIb (*P* < .001, HR = 9.766), stage III (*P* < .001, HR = 15.945), stage IV (*P* < .001, HR = 52.531), surgery (*P* < .001, HR = 0.378), chemotherapy (*P* < .001, HR = 0.639), radiation (*P* = .0058, HR = 0.745), RPS (*P* < .001, HR = 2.335), RAS (*P* < .001, HR = 1.827), RBAS (*P* < .001, HR = 3.638), LND (*P* < .001, HR = 0.497), being married (*P* < .001, HR = 0.0725), size = 4–10 cm (*P* < .001, HR = 1.950), size ≥10 cm (*P* < .001, HR = 3.303), grade II (*P* = .0011, HR = 1.653), and grade III (*P* < .001, HR = 2.007) were independent prognostic factors.

The three methods showed that age, metastasis, AJCC stage, surgery, chemotherapy, RSS, LND, tumor size, and tumor grade were prognostic factors affecting SCC survival. Meanwhile, the HR and 95% confidence interval (CI) values for age, marital status, metastasis, surgery, chemotherapy, RPS, RAS, and LND in the three methods were almost the same, whereas there were large differences in the values for stages Ib, IIa, IIb, III, and IV, RBAS, tumor size, grade II, and grade III between the three methods. For example, the HRs in the SD analysis for stages Ib, IIa, IIb, III, and IV, RBAS, tumor size, grade II, and grade III were very close to those in the CS analysis, while the values in the multivariate Cox regression analysis were lower. In contrast, the *P* values for race (Cox regression: 0.0019, SD: 0.727, CS: 0.0299) and radiation (0.005, 0.0323, and 0.0058, respectively) demonstrated that race and radiation were prognostic factors affecting survival in the Cox regression and CS analyses, which differed from the findings in the SD analysis. The *P* values for other marital status (Cox regression: <0.001, SD: 0.5268, CS: 0.2540) showed that being separated, divorced, or widowed was an independent prognostic factor in the Cox regression analysis, in contrast to in the CS and SD analyses. However, the results for grade IV (Cox regression: 0.2219, SD: 0.0.836, CS: 0.0417) showed that it was only statistically significant for the prognosis in the CS analysis.

## 4. Discussion

In this study, 5591 patients met the inclusion criteria. At the last follow-up, there were 1342 DOCs, constituting 45.96% of all deaths, which would be taken as censored data when using the conventional statistical analysis methods for survival data. Traditional statistical methods for analyzing the risk of disease include Kaplan–Meier survival analysis and Cox regression analysis, but these approaches can overestimate the CIF by failing to account for the competing risks of death.^[17,18]^ The aim of using a competing-risks model is to more accurately identify prognostic factors of SCC, when censoring is absent or when censoring is present but always observed. Competing-risks models were used in hazard function regression in the CS model and the SD models, which is also called the CIF regression model or Fine-Gray model.^[14]^ The CS model may be better suited to addressing etiological questions, since it allows the effect of covariates on the rate of occurrence of the outcome to be estimated in those subjects who are currently free of events. In contrast, the SD model may be better suited to estimating the clinical prognosis of patients, since it allows the effect of covariates on the absolute risk of the outcome to be estimated over time.^[15]^ Latouche et al pointed out that using SD and CS models simultaneously is generally the most rigorous scientific approach to analyzing competing-risks data.^[16]^ In the present study, the SD model, which focuses on the direct assessment of actual risks and therefore tends to assess disease risk and prognosis, seemed to be more valuable. Overall, the HR and 95% CI values for most variables were close in the SD and CS analyses, and the correlation direction was consistent, but fewer variables were inconsistent. It is clear that compared to the SD model, Cox regression analysis clearly overestimated the prognostic effect of certain covariates such as race, AJCC stage, metastasis, radiation, RSS, tumor size, and tumor grade.

Some studies have found that the incidence of SCC in all age groups except 0 to 24 years old remained stable from 1993 to 2012, while for the 5-year survival rate, it was higher in whites than blacks between 1983 and 1992 (70.5% vs 58.9%).^[6,7,19]^ During the three decades from 1983 to 2012, the relative risks for age were 1.045, 1.038, and 1.026, respectively, and those for race were 1.221, 1.249, and 1.186. Furthermore, even when accounting for stage, histology, and race, increasing age showed a worse overall survival ratio among the different stages. For young women aged 20 to 49 years old, aggressive treatment demonstrated a significant survival advantage compared with less-aggressive regimens or no treatment. In contrast, for women aged at least 50 years, aggressive treatment and less-aggressive therapy provided an obvious survival advantage over no treatment. Those studies indicated that age and race were independent negative prognostic factors for overall survival in cervical cancer using the Chi-square test and Cox regression, which led to statistical deviation to a certain extent. The results of Cox regression, SD, and CS analyses obtained in the present study indicated that age was a prognostic factor for DCC, but the HR was higher in the Cox regression analysis than in the SD and CS analyses, at 1.018, 1.006, and 1.009, respectively. However, the multivariate Cox regression analysis indicated that race was not a prognostic factor for cause-specific mortality, which directly contrasted with the results of the SD and CS analyses. This indicates that the multivariate Cox regression analysis overestimated the effect of race despite the sample being large, which is due to it not being applicable to the competing-risks model of deletion data.^[15]^ The results of the SD and CS analyses for the effect of race had a higher reference value.

Marital status has been considered to be an independent predictor of the tumor stage at diagnosis and survival in women with cervical cancer, and its predictive efficacy has been confirmed using multivariate logistic regression models.^[8]^ That study found that unmarried women (single, separated, divorced, or widowed) were being diagnosed more often at an advanced stage and had worse survival compared to married women in the US because they were less involved in cervical cancer screenings. Meanwhile, another study analyzed a binary logistic regression model to show that the number of single women with cervical cancer had increased significantly over the past 4 decades, especially dramatically among single women aged ≥40 years,^[10]^ which also demonstrated that improving screening strategies might help reduce the incidence of this malignancy. The three models analyzed in the present study produced the same conclusion about the effect on the prognosis of married women compared with a single marital status. However, when compared with other marital statuses including separated, divorced, or widowed, the predictive efficacies of the SD and CS analyses differed, with the two studies above and the Cox regression analysis showing that only being married was a positive independent contributing factor.

A prognostic analysis based on the SEER database found that most malignancies of the uterine cervix in single women were the squamous cell carcinoma subtype, high grade, and involved larger tumors (>4 cm).^[10]^ Tumor grade, tumor size, and International Federation of Gynecology and Obstetrics (FIGO) stage were associated with an increased risk of LN metastasis in the analysis of overall survival. The multivariate analysis of cause-specific survival and overall survival performed by Macdonald et al revealed that lower grade, lower FIGO stage, smaller tumor, fewer involved LNs, and a lower or zero positive LN ratio were independent predictors compared with para-aortic LN involvement, and positive LNs in cervical carcinoma predicted a prognosis that was inversely related to the number of involved LNs.^[20]^ Colturato et al also demonstrated that LN micro-metastasis was an important risk factor for tumor recurrence.^[9]^ More SCCs were diagnosed in younger women and were of a poor or undifferentiated grade. In contrast, more cervical adenocarcinomas presented with a well-differentiated grade and involved older women.^[21]^ Another study confirmed that increasing FIGO stage gradually decreased the coincidence rate of the two staging methods,^[22]^ and AJCC stage could more accurately reflect the lesion range than the FIGO stage. Furthermore, a negative LN count was an independent prognosis factor for patients with cervical cancer at each FIGO stage, and was a good supplement for evaluating the prognosis of the FIGO stage.^[23]^ Few studies have explored the effect of AJCC stage on the prognosis of SCC compared to the FIGO stage. In our study, AJCC stage, metastasis, tumor size, grade II, and grade III were prognostic factors for SCC in the three models, and higher AJCC stage, larger tumor, LN metastasis, and higher grade (except grade IV, which was not a prognostic factor) had significantly adverse effects on SCC.

Other studies have investigated the effects of different treatments on the SCC disease risk and prognosis using traditional Cox regression analysis. Surgery and radiation were found to be common treatments for patients with cervical cancer stages IA2–IIA. Radical hysterectomy in combination with regional lymphadenectomy is the conventional treatment for these people.^[12]^ A multivariate analysis showed that the overall survival of stage IA cervical cancer in patients younger than 50 years was significantly better among those who underwent hysterectomy and ovarian conservation compared with those who underwent oophorectomy without radiotherapy, but that the disease-specific survival was approximately the same in the two groups.^[11]^ This indicates that surgical ovarian conservation is a positive independent prognostic factor for overall survival in the early stage among young patients with cervical cancer. However, for locally advanced cervical cancer, hysterectomy and concurrent chemoradiotherapy were standard treatments, and could improve the overall survival.^[24]^ Another study noted that numerous regimens including hysterectomy prolonged the survival time in stage IIB patients but not in stage III patients with cervical cancer.^[25]^ Shah et al also pointed out that more extensive lymphadenectomy had no effect of survival among those with positive LNs, but increased survival in those with negative LNs.^[26]^ Moreover, patients with more LNs resected had a lower probability of dying from cervical cancer. A prospective study indicated that patients with cervical cancer at stages IB1–IIA1 receiving radical hysterectomy had fewer severe postoperative complications including urinary infections and/or lower limb lymphedema, and that preoperative brachytherapy was an independent risk factor for severe morbidity after surgery.^[27]^ Several studies found that neoadjuvant chemotherapy followed by surgery significantly improved the prognosis in locally advanced cervical cancer, especially for tumors larger than 4 cm.^[28–30]^ However, other studies have shown that previous research might have overestimated the treatment effect because it was unclear whether the chemotherapy doses and methods were optimal.^[31,32]^ There have been few studies of how the order of surgery and radiotherapy affects the prognosis. The multivariate analyses of the three models performed in our study revealed that undergoing surgery, chemotherapy, and LND were positive independent prognostic factors for survival. Using those treatments could reduce the cause-specific death rate by two to three times compared to no treatment. Our study has also shown that regardless of the sequence of surgery and radiotherapy, combined treatment methods could increase the mortality rate by two to four times compared to only applying surgery or radiotherapy. Our results contrast with previous reports that undergoing radiation did not significantly affect the prognosis of the SCC.

The study was limited by using the SEER database, which does not cover all possible factors impacting the patients, instead providing only some basic information. We did not further stratify the prognostic factors based on a comprehensive impact assessment. The small sample size also affects the reliability of the statistical results. In addition, whether economical status and comorbid condition of patients are prognostic factors affecting survival in patients with SCC has not been analyzed in this study, which may consider in future studies. All these factors contribute to decreasing the reliability of the conclusions drawn.

## 5. Conclusion

A competing-risks model is a new statistical method that was used in this study to more accurately identify prognostic factors when censoring was absent or when censoring was present but always observed compared to using the conventional Cox regression analysis that has commonly been used in many previous studies. We also performed conventional Cox regression, SD, and CS analyses because they had their own explanation being used as references. The SD model used in this study may be better suited to estimating the clinical prognosis of a patient, which made it possible to estimate the effect of covariates on the absolute risk of the outcome over time. Overall, the results obtained using the SD model analysis were close to those obtained in the CS analysis, but Cox regression overvalued the clinical prognosis of covariates, leading to bias in the results. We expect that the competing-risks model will be more feasible for evaluating the prognosis and guiding clinical practice in the future.

## Acknowledgments

We deeply appreciate the help from all our colleagues at the Clinical Research Center, The First Affiliated Hospital of Xi’an Jiaotong University, who offered their valuable insights.

## Author contributions

**Conceptualization:** Junyan Cao.

**Data curation:** Chengfeng Hu, Junyan Cao.

**Formal analysis:** Chengfeng Hu, Junyan Cao, Li Zeng, Yao Luo.

**Funding acquisition:** Junyan Cao.

**Investigation:** Chengfeng Hu, Junyan Cao, Li Zeng.

**Project administration:** Hongyuan Fan.

**Resources:** Hongyuan Fan.

**Software:** Yao Luo, Hongyuan Fan.
